# Technical laboratory procedures faced on many oocytes picked up *in vitro* fertilization cycles

**DOI:** 10.5935/1518-0557.20250015

**Published:** 2025

**Authors:** Christina Morishima, Nilka Fernandes Donadio, Luiz Henrique Gebrim, Tatiana Carvalho S. Bonetti

**Affiliations:** 1 Departamento de Ginecologia, Escola Paulista de Medicina, Universidade Federal de São Paulo (EPM-UNIFESP), São Paulo, Brazil; 2 Centro de Reprodução Humana Governador Mario Covas, Departamento de Obstetrícia e Ginecologia, Hospital das Clínicas da Faculdade de Medicina da Universidade de São Paulo (HCFMUSP), São Paulo, Brazil; 3 Hospital da Mulher, São Paulo, Brazil; 4 Hospital Beneficência Portuguesa, São Paulo, Brazil

**Keywords:** reproductive medicine, *in vitro* fertilization, ovarian hyperresponse, cryopreservation, embryology protocols

## Abstract

**Objective:**

The accumulation of cryopreserved embryos in assisted reproductive technology (ART) centers is an important point of discussion for the clinical and scientific community. This study conducted a survey to explore in vitro laboratories’ approaches to handling oocytes and embryos in the face of an ovarian hyperresponse.

**Methods:**

We invited ART centers whose laboratories were registered in the National Embryo Production System (2018) to answer an online multiple-choice questionnaire. A total of 57 ART centers participated.

**Results:**

Most of the ART centers were private (92.9%) and about half of them were considered medium/large business, performing at least 30 IVF cycles per month. The ART centers were asked what their concept of ovarian hyper-responsiveness was, and 76% considered more than 15-20 oocytes retrieved. Faced with an ovarian hyper-responsiveness, the main practice reported was injecting all mature oocytes and vitrifying all developed blastocysts (53%), followed by a practice of freezing half of the oocytes and injecting other half of the oocytes (35%). 9% alternated between these two protocols.

**Conclusions:**

Injection of all oocytes followed by embryos cryopreservation is the most common approach when high order number of oocytes is collected, despite it generating many surplus embryos. The findings of this study underscore the necessity for updated consensus on the management and production of embryos, considering the multifaceted considerations involved, as laboratory efficiency and costs. The decision in such cases should focus on the balance of priorities of having a healthy newborn and the responsibility for the fate of surplus embryos.

## INTRODUCTION

Reproductive medicine and assisted reproductive technology (ART) have developed intensively in scientific and technological spheres. Accompanying social transformations, the number of patients seeking reproductive assistance has been increasing annually (Zegers-Hochschild *et al.*, 2022), resulting in approximately 12 million children born through *in vitro* fertilization (IVF) in the last four decades (Adamson, 2023). At present, most infertility treatments are based on highly complex techniques. These include controlled ovarian stimulation (COS) with gonadotropins, oocyte retrieval and insemination of mature oocytes, which is carried out by intracytoplasmic sperm injection (ICSI) in over 80% of cases (Zegers-Hochschild *et al.*, 2021). The embryos produced are cultured until the cleavage or blastocyst stage, followed by subsequent evaluation and selection of the one with the highest implantation potential. The viable embryos may be transferred or cryopreserved. The COS aims to obtain many oocytes that give a higher chance of pregnancy, without patient input risks. Despite some controversies, the literature determines that 10 to 15 mature oocytes collected is the aim of a COS (Fiedler & Ezcurra, 2012; La Marca & Sunkara, 2014; Drakopoulos *et al*., 2023; Polyzos & Sunkara, 2015).

In daily practice, ovarian hyperresponses (OHR) can occur after a COS, increasing the risk of developing ovarian hyperstimulation syndrome, which is a severe adverse event. The OHR is defined as the retrieval of a higher number of oocytes than expected after COS using standard doses of gonadotropin, described by several authors as more than 15 oocytes (Drakopoulos *et al.*, 2023). The mean prevalence of OHR in IVF cycles is about 7%, but it varies according to women’s age, as 30% in women ≤ 30 years and declines with aging. However, fewer than 5% of cycles progress to moderate or severe ovarian hyperstimulation syndrome (Zeilmaker *et al*., 1984). After the introduction of GnRH agonist for triggering, the incidence of ovarian hyperstimulation syndrome is even lower (Ovarian Stimulation TEGGO, 2020; Vaughan *et al.*, 2017). Anyway, the cryopreservation of gametes and embryos plays a fundamental role in the management of patients in OHR with or without risk of ovarian hyperstimulation syndrome.

From a laboratory point of view, cryopreservation techniques allow us to manage procedures in the event of a large number of recovered oocytes, benefiting the integration of clinical care with the personal needs of patients with a personalized and multi-professional approach (Practice Committee of the American Society for Reproductive Medicine, 2016). Several laboratory strategies are possible. One such approach involves inseminating all mature oocytes, potentially generating numerous embryos, with all viable embryos cryopreserved, called freeze-all strategy (Timmons *et al.*, 2019; Abreu *et al.*, 2021). Oocyte vitrification is also currently considered a highly effective option, although it is a less commonly employed approach (Paynter *et al.*, 1999; Smith *et al.*, 2010; Stoop *et al*., 2011). The practice of fertilizing some oocytes and cryopreserving part of them is also an option. Regardless of the approach adopted, if too many oocytes are inseminated, many surplus embryos are potentially produced, which leads to many practical challenges, and ethical and legal conflicts. The growing need for physical storage space and efficient traceability systems for cryopreserved embryos is a reality in most ART centers (Scott *et al*., 2013). On the other hand, the ethical and legal conflicts related to oocyte cryopreservation are less complex, making management more user-friendly (Ubaldi *et al.*, 2010). The desire for maximal oocyte recruitment to ensure the possibility of embryo transfer and a higher cumulative pregnancy rate per cycle, condemns us to the custody of surplus embryos, prompting the need for discussion regarding the fate of cryopreserved embryos (Fuscaldo *et al*., 2007). Another common situation is the search for a greater number of embryos produced for pre-implantation genetic test for aneuploidies (PGT-A). Since it is assumed that some of these embryos will be aneuploid and ineligible for transfer, a greater number of embryos produced would guarantee a greater chance of at least one euploid embryo for transfer. But at the cost of many embryos produced and cryopreserved (Practice Committees of the American Society for Reproductive Medicine & the Society for Assisted Reproductive Technology, 2018).

The embryo production in Brazilian clinics is monitored by the National Health Surveillance Agency (*Agência Nacional de Vigilância Sanitária* - ANVISA). All ART clinics report the laboratory indicators for embryo production for the National Embryo Production System (*Sistema Nacional de Produção de Embriões* - SisEmbrio) since 2008. Based on those reports, there were 13% increase in the number of embryos cryopreserved between 2012 and 2019, reaching more than 100,000 embryos stored/year. In 2020 there was a decrease of ≈35% (85,448), probably reflecting the COVID-19 pandemic. However, the number of cryopreserved embryos has risen again since then. In 2022, 297,848 embryos were cryopreserved, representing a 33.7% increase compared to 2019 (SisEmbrio - ANVISA, 2022). Similarly, data from the Latin American Network for Assisted Reproduction (*Red Latinoamericana de Reproducción Asistida* - REDLARA), which reports data from all Latin American countries, including Brazil, follows the same pattern and shows a progressive increase in the number of frozen-thawed embryo transfers, from 1,677 in 2012 to 28,184 in 2019 (Zegers-Hochschild *et al.*, 2022).

The fate of surplus embryos is a sensitive issue. Countries like Australia, the United States, and Switzerland allow for discarding surplus embryos, as well as the practice of donation for the treatment of other couples or scientific research (Mohler-Kuo *et al.*, 2009; Jin *et al.*, 2013; SisEmbrio - ANVISA, 2022). In Brazil, the Federal Council of Medicine (CFM) regulates the ethical standards of human reproduction, and some changes have taken place in recent years. In 2021, the regulations on donation for research, to another couple or embryo disposal were updated. The disposal of embryos after three years of storage is now only allowed with judicial authorization, even if authorized by the couple. In addition, it was established that a maximum of 8 embryos could be produced and frozen in one IVF cycle (CFM, 2021). However, these new regulations made it impossible to personalize treatment in the face of the many variables of infertility and ART. Consequently, a new update was published about a year later, and it is established in our country: 1. there is no limit on maximum embryos to be produced, 2. the fate of surplus embryos is a shared decision of each clinic/medical doctor with the patient or couple (CFM, 2022).

The accumulation and abandonment of cryopreserved embryos are important points of discussion for the clinical and scientific community. However, their importance is outweighed by the chances of pregnancy with the number of embryos produced in a treatment cycle. On the other hand, laboratory management of many oocytes can minimize complications related to the accumulation of embryos. This study aimed to examine the main processes used in IVF laboratories concerning the management of oocytes and embryos in the face of OHR, with many oocytes, in ART clinics in Brazil.

## MATERIAL AND METHODS

This was a cross-sectional study based on a questionnaire, applied to IVF laboratories coordinators from the ART clinics in Brazil. The study was developed in the Department of Gynecology of the *Escola Paulista de Medicina - Universidade Federal de São Paulo* (EPM-UNIFESP). The research protocol was approved by the UNIFESP Research Ethics Committee (CAAE: 16371419.0.0000.5505). All participants signed the informed consent term previously to the questionnaire answering.

### Questionnaire construction and validation

The questionnaire was designed to explore the IVF laboratories’ practices, specifically, in managing oocytes and embryos within the context of a high number of oocytes collected after a COS. The questionnaire was organized into three sections: (i) the pre-laboratory phase, encompassing profiles of the ART clinics; (ii) the laboratory phase, focusing on technical-operational procedures; and (iii) the post-laboratory phase, aimed at understanding specimen utilization and storage, including cryopreserved supernumerary oocytes and embryos.

The development and validation of the questionnaire occurred in two phases. Initially, the first version of the questionnaire was prepared by a team composed of senior’ clinicians and embryologists. Five IVF laboratory coordinators were invited to answer the questionnaire as part of the validation process. Four IVF laboratory coordinators responded, providing valuable insights, identifying challenges, and offering suggestions for improvement. After the adjustments based on Phase I, the revised questionnaire was administered to an additional 10 randomly selected IVF laboratories coordinators, as part of the second phase of questionnaire validation. In the second phase, we aimed to validate the final version and to evaluate the efficiency of the research process, as time required for completion and feasibility of the electronic system utilized. Nine IVF laboratories participated in the second phase of questionnaire validation and the final version of the questionnaire comprised 14 multiple-choice questions, into three sections: (i) demographics (two questions); (ii) laboratory / technical (seven questions); and (iii) post-laboratory (five questions).

The online research platform SURVIO was used for questionnaire administration and data collection, automatically tabulating response frequencies while preserving respondent anonymity through an automatic warning mechanism that obscured sender identities. Visits to the questionnaire were exclusively via direct link.

### Participants

The 11^th^ report of the SisEmbrio included a list of 146 Brazilian IVF clinics. Among those, nine clinics were affiliated services of a principal clinic, and three services ceased operations during the study period. Then, we considered 134 ART clinics as possible participants of this study, in Brazil. Aiming to attain as many IVF centers as possible, we used the snowball sampling method. It started with some study participants, found by personal contacts of the researcher. Afterward, each IVF laboratory coordinator was asked to refer three other possible participants, and the process continued until we reached a saturation point.

The invitations were initially made via voice calls, in which a brief presentation of the study was given to the IVF laboratory coordinator. Formal invitations were sent by email, accompanied by a presentation of the study, the informed consent form, and a link to access the questionnaire. We invited 94 IVF clinics to take part, using the snowball sampling method. Another 40 centers were contacted through customer service channels, such as email and phone calls, without success. The response period was from November 2020 to May 2021.

**Figure 1:**
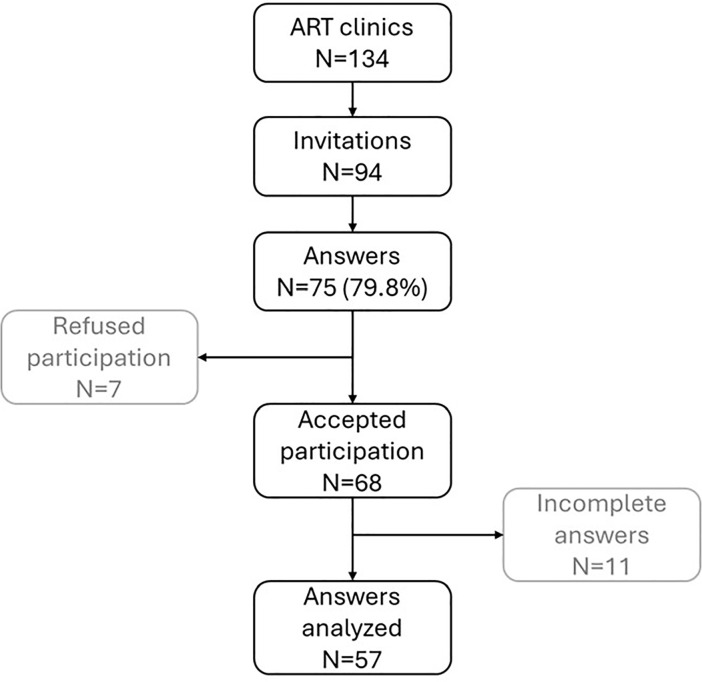
Study design.

The overall response rate was 80% (75 responses out of 94 invitations). However, seven responses were declinations to participate and consequently, 68 responses were considered. We excluded the other 11 questionnaires due to incomplete data, resulting in 57 valid responses for analysis in the study (Figure 1).

### Data analysis

The data generated by the SURVIO system was tabulated in an Excel spreadsheet and then analyzed using the Jamovi Software (V. 2.3). Demographic data and questionnaire responses were presented by frequency and percentiles and correlations between them were analyzed. Means were compared by student’s t-test and ANOVA, and proportions by chi-square or Fisher’s exact tests, as appropriate. Values of *p*<0.05 were considered statistically significant.

## RESULTS

The 57 valid responses were evaluated. The demographic questions asked the centers about administration model (public or private), productivity levels (small-business: up to 30 IVF cycles per month, medium/large-business: more than 30 cycles monthly), and experience in cryopreservation techniques (expert: over 5 years of experience, non-expert: less than 5 years of experience). Most of the centers participating were private and had over 5-year expertise in cryopreservation techniques. About 50% of centers were medium/large-business and 50% were small-business (Table 1).

**Table 1:** Demographic characteristics of the participating IVF centers.

Characteristics	N (%)
**Administration model**	
Public	7.0%
Private	93.0%
	
**Productivity levels**	
small business: up to 30 IVF cycles/month	48.0%
medium/large business: more than 30 IVF cycles/month	52.0%
	
**Experience in cryopreservation techniques**	
non-expert: less than 5 years of experience	19.0%
expert: over 5 years of experience,	81.0%

**Figure 2:**
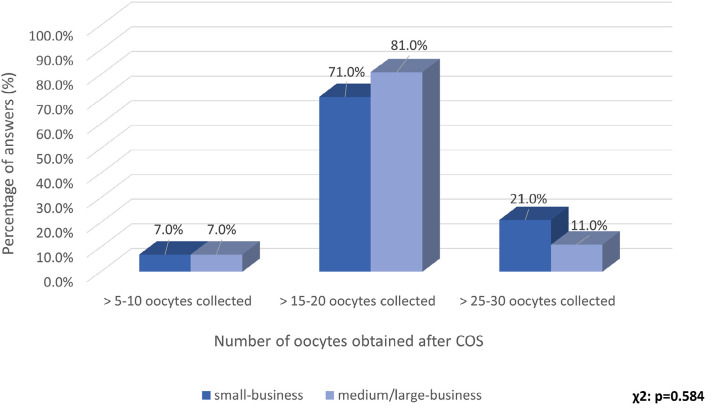
Responses on the definition of OHR, by the number of oocytes collected in the participating centers. The responses were presented according to the productivity of the IVF center.

Since there is no consensus on the definition of ovarian hyperresponse, and this concept could be associated with the subsequent stages of laboratory management of oocytes and embryos, participants were asked about the definition adopted at the IVF center. The responses were categorized into three ranges: 5-10, 15-20, or 25-30 oocytes recovered. Most of the IVF centers adhered to the definition of hyperresponse as over than 15-20 oocytes recovered in a stimulated cycle, in line with the most of literature data (La Marca & Sunkara, 2014). The responses were also evaluated according to the productivity of the IVF center, and there was no difference in the response profile (Figure 2).

### Laboratory procedures questions

The questions on the laboratory procedures faced by an OHR referred to the decision-making process between the doctor and the patient regarding the cryopreservation of oocytes and/or embryos and the most used protocols. The first question asked if there had been any discussions with the patients, about their interest in storing embryos or gametes, before the IVF cycle started. All respondents said it was discussed with all patients before treatment initiation. Then, we asked what approach would be most chosen if patients expressed a desire not to cryopreserve. Around half of the clinics reported individualizing behaviors based on shared decision-making between the clinician and the patient (53%), while 37% reported offering a mild ovarian stimulation protocol to reduce follicular recruitment, and the remaining centers (10%) stated they did not encounter such situations. Subsequently, we asked IVF centers to indicate the protocol that best reflected their routine practices in case of an OHR and presented five options (Box 1). When “Protocol 5: Shared decision”, was chosen, respondents were asked to describe which protocol was most frequently used. These answers were evaluated individually and allocated to the options presented in Protocols 1 to 4, and the analysis was carried out considering those four options.

**Box 1:** Protocols proposed for how the laboratory should handle oocytes and/or embryos in cases of ovarian hyperresponse.

Protocol 1: 100%-OV	All mature oocytes are vitrified
Protocol 2: DESC/ICSI	Part of the mature oocytes are discarded, and the remaining parts are subjected to ICSI. The embryos generated are vitrified.
Protocol 3: 50/50-OV/ICSI	Part of the mature oocytes are vitrified, and the remaining parts are subjected to ICSI. The embryos generated are vitrified
Protocol 4: 100%-ICSI	All mature oocytes are submitted to ICSI. The embryos generated are vitrified
Protocol 5: Shared decision	Shared decision-making between the clinician and the patient

**Figure 3:**
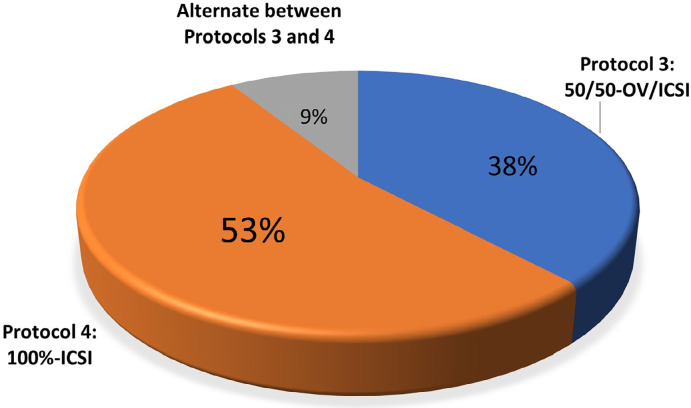
Frequencies of laboratory protocol used by ART clinics faced ovarian hyperresponse. Protocol 3: 50/50-OV/ICSI - Part of the mature oocytes are vitrified, and the remaining part is subjected to ICSI. The embryos generated are vitrified. Protocol 4: 100%-ICSI - All mature oocytes are submitted to ICSI. The embryos generated are vitrified.

The most frequent protocol cited was “Protocol 4: 100%-ICSI”, where all mature oocytes are submitted to ICSI, and embryos are vitrified. The second most common protocol cited was “Protocol 3: 50/50-OV/ICSI”, which involved fractionating the mature oocytes, part of them is vitrified, the remaining part is subjected to ICSI, and embryos generated are vitrified. The remaining 9% of centers were described to alternate equally among Protocols 3 and 4. None of the participating centers cited total vitrification of retrieved oocytes or partial disposal as the primary measures adopted. Only one center mentioned suggesting the donation of a portion of the oocytes (Figure 3).

### Scenarios regarding surplus cryopreserved oocytes and embryos

**Figure 4:**
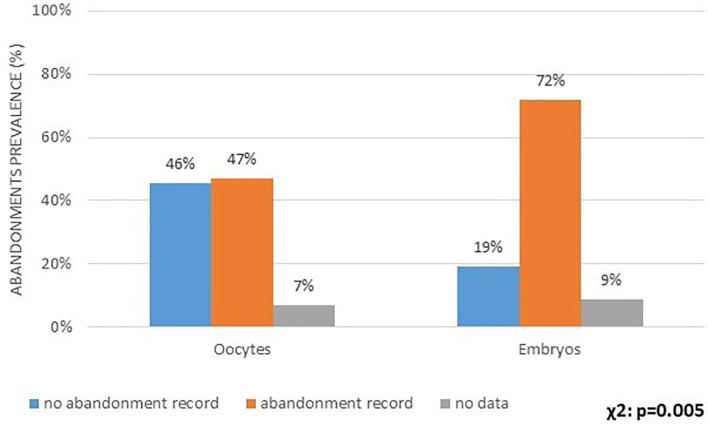
Incidence of ART centers reporting embryo and oocyte abandonment.

To explore the scenarios related to the storage of oocytes and embryos, such as abandonment and storage time, we asked the centers what definition of embryo abandonment (CFM, 2022). Most of the centers (63%) consider embryo abandonment as the “loss of contact with the patient, without traceability, for more than one year”, followed by “lack of financial responsibility after one year” (26%). The “other” option accounted for 11%. We then asked if the IVF centers experienced embryo and/or oocyte abandonment. Both situations were declared, but embryo abandonment prevalence is more frequent than oocyte abandonment (Figure 4). Less than 10% of centers declared do not have access to that information.

### Factors influencing decisions regarding conduct in the face of ovarian hyperresponse

**Figure 5:**
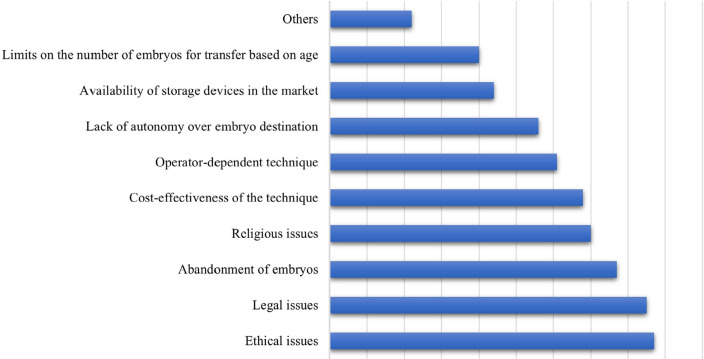
Rank of factors, from most to least importance in their association with the OHR decision-making process.

Participants were questioned on which factors influenced the decision-making process about the protocols adopted in the face of OHR. Options included ethical, legal, and religious issues, abandonment of embryos, cost-effectiveness of the technique, operator-dependent technique, lack of autonomy over embryo destination, availability of storage devices in the market, limits on the number of embryos for transfer based on age, and other factors. Respondents were asked to rank these factors from most to least importance in their association with the OHR decision-making process. Based on these responses, ethical issues emerged as the most significant factor cited by the participants, followed by legal, embryo abandonment, and religious (Figure 5).

## DISCUSSION

The main objective of this study was to outline the profile of IVF laboratories in assisted reproduction centers throughout Brazil concerning the management of oocytes and embryos in cases of OHR. The study was based on a representative sample of Brazilian IVF centers, with a response rate of 68% of invitations, representing 43% of all centers registered with SisEmbrio at the time of the study. The questionnaire applied assessed aspects ranging from the administrative structure of the center, laboratory conduct in the face of OHR, and factors associated with the decision-making process about large numbers of oocytes and embryos.

In line with the Brazilian IVF clinics profile, most of the participants had a private administrative system, and medium/large productivity (>30 cycles per month) represented about half of the interviewed centers. Regardless of economic activity or productivity, most IVF laboratories have more than five years of experience in cryopreservation, which is a crucial factor in evaluating the protocols for many oocyte management. The definition of OHR admitted by the IVF centers is another high-importance point as it directly affects the conduct. Most centers agree with the literature and consider 15-20 oocytes recovered after ovarian stimulation with standard doses of FSH as an OHR (Drakopoulos *et al.*, 2023). Interestingly, a significant percentage (≈15%) of centers consider OHR only when more than 25 oocytes are recovered. This approach is challenging, as it can lead to a greater risk of OHSS, as well as possibly generating many surplus embryos if adaptations to the protocols for oocyte management are not performed.

Regarding protocols managing many oocytes in cases of OHR, it became clear that Brazilian assisted reproduction centers employ two main approaches. A little more than half of the centers opt to inject all the recovered oocytes (100%-ICSI), and fractional oocyte fertilization has emerged as the second most used protocol (50/50 OV-ICSI). Notably, the vitrification of all oocytes protocol is not adopted in Brazilian clinics, despite the oocyte vitrification efficacy has been demonstrated in the literature (Sciorio *et al*., 2023; Hirsch *et al.*, 2024). Despite of vitrification of all oocytes protocol or 50/50 OV-ICSI being a more rational approach to avoid producing many surplus embryos, 100%-ICSI is the most used protocol.

In this study, it was not possible to establish why the 100% ICSI protocol is the most widely used. However, we can assume that it is due to the fear of a decline in success after thawing. A recent review showed that the overall success rate after planned oocyte cryopreservation decreases with the age of cryopreservation (Vaughan *et al.*, 2017; Sciorio *et al*., 2023; Drakopoulos *et al.*, 2016) which is in line with this possible fear of some clinics. Although it was not a direct question in our study, this protocol may have been chosen so often to carry out the genetic study of the embryos (PGT-A) (Practice Committees of the American Society for Reproductive Medicine & the Society for Assisted Reproductive Technology, 2018). On the other hand, the 100%-ICSI protocol, most used by Brazilian clinics, results in the formation of surplus embryos, which can become a difficult management situation.

It therefore seems rational to us that the 50/50 OV-ICSI protocol is the most suitable for balancing costs and benefits. Considering those two main protocols practiced by Brazilian IVF clinics, we simulated the financial cost per cycle for OHR cases, from the laboratory’s point of view, considering the success rates expected for these procedures. For both the 100%-ICSI and 50/50 OV-ICSI protocols, we considered that 15 oocytes were supposed to be collected, which represents the limit accepted in the literature (Drakopoulos *et al.*, 2023; La Marca & Sunkara, 2014) and by most clinics interviewed in this study, as the limited number of oocytes to define OHR. We used references from ALPHA Scientists in Reproductive Medicine (ESHRE Special Interest Group of Embryology & Alpha Scientists in Reproductive Medicine, 2017) to calculate the rates of mature oocyte retrieval, normal fertilization, cleavage, blastocyst formation, embryo survival, and oocyte survival. For success rate, we used data reports by Society for Assisted Reproductive Technology (SART, 2019). In addition, we used the values of laboratory supplies practiced on the Brazilian market in the year 2023. After the calculations, the final values were converted into dollars using the rate U$ 1.0 = R$ 5,20.

**Figure 6:**
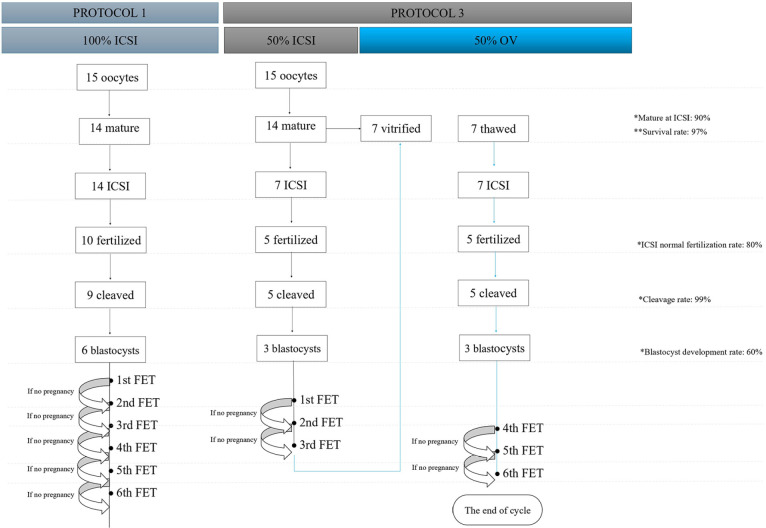
Diagram calculating the approximate average cost of freezing all (100%-ICSI) and partially fertilizing the oocytes (50/50 OV-ICSI).

For cycles that perform 100% ICSI, starting from 15 oocytes, we assume the formation of six blastocysts (fertilization rate of 75%, and blastocyst formation rate of 50%), allowing for six single embryo transfers. The average laboratory cost for this protocol is approximately U$ 900.00 (R$ 4,700.00). If we estimate that we have a success rate (live births) of 30% per transfer, after three transfers we would achieve around 70% of success. It is therefore estimated that until five surplus embryos would remain cryopreserved in these cases (Figure 6).

In cases of 50/50 OV-ICSI, we consider that eight mature oocytes are vitrified and seven are submitted to ICSI, resulting in three viable blastocysts, at an average cost of approximately U$ 640.00 (R$ 3,335.00). If we consider the same success rates of 30% live births in three single embryo transfers, we will achieve success in 60-70% of cases, and a maximum of two surplus embryos could be maintained cryopreserved. In the remaining 30-40% of cases, it may be necessary to thaw the vitrified oocytes and then perform ICSI, resulting in an additional cost of approximately U$ 640.00 (R$ 3,335.00). In this last situation, the average accumulated laboratory cost would be approximately U$ 1280.00 (R$ 6,670.00) (Figure 6). We can observe that the cost of 50/50 OV-ICSI protocol is lower than 100% ICSI for the most of cycles (around 60-70%) e more expensive for 30-40% of cases. But it has the advantage of a smaller number of surplus embryos cryopreserved.

It is interesting to note that there is a conflict between laboratory conduct in OHR and responses to factors influencing decision-making processes. While almost all of the center’s report experiencing embryo abandonment, and cite ethics, legislation, and embryo abandonment as the main influencers of decision-making concerning the large number of oocytes collected, the main conduct (100% ICSI) leads to the formation of many surplus embryos. The complexity of decision-making processes in ARTs extends beyond clinical and laboratory considerations, encompassing ethical, legal, and cost-effectiveness factors. The findings of this study underscore the necessity for updated consensus on the management and production of embryos, considering the multifaceted considerations involved. The absence of clear guidelines regarding embryo disposition highlights the need for regulatory bodies to comprehensively address ethical, legal, and moral concerns. Clarification and standardization of protocols are essential to ensure responsible and effective ART practices.

Concerns about surplus embryos and abandonment persist, despite attempts to establish guidelines. The need for more studies evaluating the cost-effectiveness of technical laboratory protocols as individualized medical protocols become more and more prevalent is highlighted by societies, but there is still no definite consensus. This study provides some relevant points to reflect on the decision-making process in OHR:

i. The main protocol used by clinics is the one that generates the largest number of surplus embryos possible
ii. The financial cost of the protocols is not relevant enough to justify this choice for 100%ICSI or 50/50 OV-ICSI
iii. Embryo abandonment is a reality and a concern in most IVF centers, but the procedures used in OHR are not aligned to avoid this situation

This study has its limitations. We did not extend our approach to genetic evaluations of the embryos, which is currently common practice in IVF treatments (Argyle *et al*., 2016) and may be associated with the choice of protocols in OHR. Submission of all oocytes to ICSI may be a choice to obtain more embryos to be biopsied for genetic analysis and increase the chances of having euploid embryos for transfer (Greco *et al.*, 2020). We understand that individualized choices of protocols for managing large numbers of oocytes in cases of OHR should consider whether or not a genetic analysis of the embryos has been carried out and the age of the patients, which are directly related to the prevalence of aneuploidy (Capalbo *et al.*, 2017) and success after oocyte thawing (Hirsch *et al.*, 2024).

In summary, we can conclude that the protocol for injecting all the recovered mature oocytes and then cryopreserving all the viable embryos (100% ICSI) is the most widely used in Brazilian clinics, followed by the protocol where half the mature oocytes are vitrified and the other half are injected, with cryopreservation of the viable embryos. Embryo abandonment is a reality in the vast majority of clinics (70%) and ethical issues, legislation, and embryo abandonment are the factors considered most important in the decision-making process in cases of OHR. Despite this, the most prevalent approach is not one that avoids a large number of surplus embryos.
